# Hierarchical Factor Analysis and Factorial Invariance of the Chinese Overparenting Scale

**DOI:** 10.3389/fpsyg.2019.01873

**Published:** 2019-08-14

**Authors:** Janet T. Y. Leung, Daniel T. L. Shek

**Affiliations:** Department of Applied Social Sciences, The Hong Kong Polytechnic University, Hong Kong, China

**Keywords:** overparenting, early adolescents, confirmatory factor analysis, hierarchical factor analysis, factorial invariance, Chinese

## Abstract

Overparenting has become an emergent phenomenon, where parents intrude into the lives and directions of their children and remove any anticipated obstacles that their children may encounter. This phenomenon develops rapidly across different ages, nations and cultures. This study examined the dimensionality of the Chinese paternal/maternal overparenting scales (CPOS and CMOS) in 1,735 early adolescents (mean age = 12.63 ± 0.78 years; 47.4% were female) in Hong Kong. Confirmatory factor analyses indicated that an 8-factor model fitted the data well for both scales. The factors included close monitoring, intrusion of child’s life and direction, over-emphasis on child’s academic performance, frequent comparison of child’s achievement with others, overscheduling of child’s daily routine, anticipatory problem-solving, excessive affective response and excessive care. Hierarchical factor analyses showed that these factors could be subsumed under two second-order factors of “over-demandingness” and “over-responsiveness,” which provides support for the conceptual framework of parenting. Furthermore, the hierarchical factor models of the CPOS and CMOS were invariant in adolescent boys and girls; the scales and subscales showed good internal consistency. The present findings suggest that the CPOS and CMOS showed good factorial validity and reliability that can be used to assess overparenting objectively among early adolescents in the Chinese contexts.

## Introduction

During the past two decades, overparenting has become an emergent phenomenon that draws the attention of researchers, mass media and the public (e.g., Gibbs, 2009; Segrin et al., 2013). Overparenting can be conceived as a developmentally inappropriate parenting style where parents intrude into the lives and directions of their children and remove any anticipated obstacles that their children may encounter to safeguard the success and happiness of their children (Segrin et al., 2012). Rousseau and Scharf (2015) further argued that overparenting imposes inappropriate levels of parental control and assistance to their offspring, which hinders their children’s desire for autonomy and self-development. Segrin et al. (2012, 2013) proposed four unique features of overparenting, including “anticipatory problem-solving and risk aversion,” “excessive advice and affective involvement,” “control over children’s self-direction,” and “provision of abundant tangible assistance.” Overparenting has become an emergent parenting style that develops rapidly at different ages, nations and cultures (Gibbs, 2009; Leung and Busiol, 2016). There is growing evidence showing that overparenting is associated with poorer self-efficacy, problem-solving capacities and interpersonal sensitivity (Schiffrin et al., 2014; Reed et al., 2016; Scharf et al., 2017), but increased narcissistic behavior of late adolescents (Segrin et al., 2012).

Though there is growing attention on research pertinent to overparenting, it is still at its infancy. Particularly, researches examining the impacts of overparenting at childhood and early adolescence are severely lacking. During adolescence, puberty as well as formation of formal operational thought have marked profound physiological and cognitive changes among early adolescents (Steinberg and Morris, 2001). Adolescents develop their self-identity, competencies and connections with the outside world (Erikson, 1968), but self-destructive and problem behavior may arise and accelerate at the same time (Elliott, 2009). According to the separation-individuation theory (Grotevant and Cooper, 1986), adolescents seek for greater autonomy and individuality from their parents, and parents may need to renegotiate relationship boundaries with their children (Longmore et al., 2013). This is a challenge for parents who worry about the potential risks and failures that their children may face in their developmental paths. Rather than granting more autonomy for adolescents to learn from trials and errors, some parents tend to over-protect their children by removing the obstacles for their children and intruding into their children’s daily routine and life paths (Padilla-Walker and Nelson, 2012). However, overparenting may imply an imbalance of parent-child role differentiation, which may hinder adolescent psychosocial development (Gavazzi and Sabatelli, 1990).

Besides, there is a severe lack of validated tools for assessing overparenting in early adolescents, which contributes to one of the barriers in conducting research in early adolescents. As most assessment tools on overparenting and helicopter parenting were validated in samples of emerging adults or their parents (e.g., LeMoyne and Buchanan, 2011; Segrin et al., 2012; Odenweller et al., 2014; Leung and Shek, 2018), there is a need to validate assessment tools on overparenting in non-Western young adolescents to see whether the features of overparenting are applicable for early adolescents.

Another issue related to overparenting is how the concepts are linked to the conceptual framework of parenting style. Parenting style has been defined as “a constellation of attitudes toward the child that are communicated to the child and that, taken together, create an emotional climate in which the parents’ behaviors are expressed” (Darling and Steinberg, 1993, p. 488). Maccoby and Martin (1983) identified two dimensions of parenting style, namely parental demandingness and parental responsiveness. While parental demandingness refers to the firmness and restrictions of the parents in supervising and controlling their children, parental responsiveness suggests warmth, support and encouragement to their children in response to their developmental needs (Maccoby and Martin, 1983). Based on different combinations of parental demandingness and responsiveness, Baumrind (1991) classified three parenting styles that are qualitatively different, namely, authoritarian, authoritative and permissive parenting. Authoritarian parents supervise their children strictly with limited support and warmth to their children’s needs. Authoritative parents are firm in supervising their children but respond to the needs of children with warmth and encouragement. Permissive parents show little control over their children but allow them to act freely according to their children’s will (Baumrind, 1991). Although Chinese parenting has been described as authoritarian and controlling (Chiu, 1987; Pong et al., 2010), recent studies showed that Chinese mothers can exercise high levels of control and support at the same time (Cheung and McBride-Chang, 2008; Shek, 2008). Overparenting, by its nature, is related to “excessive” parental demandingness and responsiveness by means of intrusion and overinvolvement (Locke et al., 2012). However, based on the qualitative data collected from psychologists and school counselors, Locke et al. (2012) found that there was no consensus among participants that overparenting has high levels of demandingness, though high levels of responsiveness were observed by the participants. Nevertheless, the study did not invite adolescents and/or parents as the participants. From a previous study of the authors, some features of Chinese overparenting, including “close monitoring,” “intrusion of child’s life and direction,” “over-emphasis of child’s academic performance,” “frequent comparison of child’s achievement with others” and “overscheduling of child’s daily routine,” were strongly related to measures of psychological control but showed a weak link with measures of parental support, whereas other features of Chinese overparenting, including “anticipatory problem-solving,” “excessive affective response” and “excessive care,” were strongly associated with parental support but weakly linked to psychological control (Leung and Shek, 2018). Hence, there is a need to examine the features of overparenting with reference to the concepts of parental over-demandingness and over-responsiveness.

Moreover, majority of the validation studies on parenting (overparenting) seldom addressed gender of the parents (e.g., LeMoyne and Buchanan, 2011; Segrin et al., 2012). However, based on the gender role theory (Eagly and Wood, 1999), fathers and mothers take up different roles in the family, which may affect their parenting styles. In the Chinese culture, fathers take up more supervisory and disciplinary role in regulating the behavior of their children, whereas mothers take up more caring role in managing the routine of their children and responding to their children’s needs (Shek, 2002). A “strict fathers, kind mothers” hypothesis (Wilson, 1974) was proposed in the Chinese families. However, this hypothesis was challenged in these two decades when mothers took up more controlling roles in the family, which results in a “strict mothers, kind fathers” or even “stricter and kinder mothers with detached fathers” phenomenon (Shek, 2008, p. 678). Obviously, due to the different parenting styles of fathers and mothers perceived by adolescents, there is a need to examine the psychometric properties of scales assessing paternal overparenting and maternal overparenting separately.

Furthermore, many validation studies did not examine whether boys and girls had different perceptions of overparenting (e.g., LeMoyne and Buchanan, 2011; Odenweller et al., 2014). However, Byrne and Shavelson (1987) suggested that any interpretations of mean differences in a measurement across gender are indeed problematic unless the underlying measuring construct exhibits the same structure in the two groups. As previous studies showed that Asian girls were more sensitive to maternal affective responses and authoritarian parenting style (Radziszewska et al., 1996; Shek, 2008), it is essential to examine the factorial invariance of paternal and maternal overparenting scales between adolescent boys and girls.

Finally, there are few studies on overparenting in non-Western contexts. With specific reference to the Chinese culture, it is anticipated that Chinese overparenting may be different from overparenting identified from the United States culture, as Chinese parenting that focuses on collectivism, familism and interdependence which are distinctive from the Western parenting that emphasize individuality, autonomy and independence (Shek, 2006). For example, it is not astonishing to reveal that Chinese parents ask for special privilege for their adolescent children in schools, fill up the sparse time of their children with tutorial classes, and make every decision for their children (Leung et al., 2018). As such, Leung et al. (2018) conducted a qualitative study of parents and adolescents to understand their perceptions and experiences of overparenting in Hong Kong. Eight features were identified from the study, including “close monitoring,” “intrusion of child’s life and direction,” “over-emphasis on child’s academic performance,” “frequent comparison of child’s achievement with others,” “overscheduling of child’s daily routine,” “anticipatory problem-solving,” “excessive affective response,” and “excessive care” (Leung et al., 2018). While some of these features resemble the conceptions of overparenting suggested by Segrin et al. (2012), some are unique to Chinese people. For instance, “anticipatory problem solving” was identified in both Chinese and the United States cultures, whereas “over-emphasis on child’s academic performance” and “frequent comparison of child’s achievement with others” appeared to be unique in the Chinese culture. Chinese parents pay more attention on adolescent academic performance and achievement, which represent family pride and honor (Chao and Sue, 1996). Moreover, “close monitoring,” “intrusion of child’s life and direction,” and “overscheduling of child’s daily routine” correspond to “parental domination of children’s self-direction,” while “excessive affective response” and “excessive care” reflect “parental disproportionate affection” and “abundant assistance to their children.” Based on the eight dimensions, Leung and Shek (2018) developed the Chinese Overparenting Scale which showed good internal consistency, test-retest reliability, convergent validity and factorial validity in a sample of Chinese college adolescents in Hong Kong. As there are few related studies based on early adolescents, we attempted to examine the psychometric properties of the measures for early adolescents.

Against this background, the current study attempted to examine the factor structure of Chinese paternal/maternal overparenting scales (CPOS and CMOS) in a sample of early adolescents in Hong Kong. Exploratory factor analyses and confirmatory factor analyses were employed to examine the dimensions underlying the conceptual model (Leung and Shek, 2018). With reference to the conceptual framework of parenting styles suggested by Maccoby and Martin (1983), the measurements were then assessed by hierarchical factor analyses to see whether the second-order factors of “over-demandingness” and “over-responsiveness” were confirmed in the tested model. Furthermore, we tested whether both CPOS and CMOS were invariant between adolescent boys and girls. Finally, we examined the internal consistency of the total scales and the subscales.

## Materials and Methods

### Participants and Procedure

Secondary school students studying in Grade 7 were recruited in the study. We adopted the multi-stage stratified cluster sampling method to recruit secondary school students, with geographical area and school banding as the stratifying factors. A total of 19 secondary schools across Hong Kong participated in the study, with 1,950 Grade 7 students joining the study. Finally, 1,735 respondents participated in the study, with a response rate of 89.0%.

Among the respondents, 912 (52.6%) were boys and 823 (47.4%) were girls. The mean age was 12.63 (*SD* = 0.78); 367 (21.2%) students came from non-intact families (remarried, divorced, separated, and widowed); 324 (18.7%) students received Comprehensive Social Security Assistance (CSSA), which is a means-tested public assistance provided by the Hong Kong Government to help the poor families; 419 (24.1%) were the only children; 864 (49.8%) students had one sibling and 296 (17.1%) had two siblings.

Written informed consent was obtained from the parents and adolescents prior to data collection. Data collection was conducted in the classroom. Trained research assistants introduced the research purpose, data collection procedure and rights on voluntary participation and withdrawal to the students. The students were invited to complete a questionnaire that contained CPOS, CMOS and questions on demographic characteristics. Those students who did not participate in the students were allowed to do their homework assignments in class. The students were given adequate time to fill out the questionnaires. We obtained ethical approval from the Human Subjects Ethics Sub-committee of an internationally recognized university.

### Measures

#### Chinese Overparenting Scale (CPOS/CMOS)

Based on the literature on overparenting (e.g., Segrin et al., 2012) and the qualitative study from focus groups of Chinese parents and adolescents in Hong Kong (Leung et al., 2018), a 42-item Chinese Overparenting Scale was developed by Leung and Shek (2018) with eight dimensions: close monitoring, intrusion of child’s life and direction, over-emphasis on child’s academic performance, frequent comparison of child’s achievement with others, overscheduling of child’s daily routine, anticipatory problem-solving, excessive affective response and excessive care. As previous CPOS/CMOS was developed for college students, four items were modified to ensure that the measurements were applicable to early adolescents. The items included Item 11: “Father/Mother makes decisions in my study and work” was changed to “Father/Mother makes decisions in my study”; Item 13: “Father/Mother tries every effort to raise my academic result” was changed to “Father/Mother tries every effort to raise my performance in school examination”; Item 15: “Father/Mother pays great attention to my examination” was changed to “Father/Mother pays great attention to my school examination”; and Item 16: “My academic report is my father’s/mother’s performance report” was changed to “My school report is my father’s/mother’s performance report.” Each item is rated on a 6-point Likert scale (from 1 = “Strongly disagree” to 6 = “Strongly agree”). The sample item of each dimension is shown in Table 1. The related measures showed good internal consistency, test-retest reliability, convergent validity and factorial validity in a sample of college adolescents in Hong Kong (Leung and Shek, 2018). Higher scores indicate higher levels of perceived paternal and maternal overparenting. Both CPOS and CMOS showed good internal consistency in this study (CPOS: α = 0.95; CMOS: α = 0.96).

**TABLE 1:** Sample item of each dimension of Chinese paternal/maternal overparenting scales.

**Dimension**	**No. of items**	**Sample item**
Close monitoring	1–4	Item 4: My father/mother always tracks my whereabouts.
Intrusion of child’s life and direction	5–10	Item 9: My father/mother requires me to follow his/her way of my development.
Overemphasis of child’s academic performance	11–15	Item 14: Whenever I have exams, my father/mother will be ready in full battle array.
Frequent comparison of child’s achievement with peers	16–20	Item 16: My father/mother always compares me with my peers.
Anticipatory problem solving	21–26	Item 23: My father/mother will eliminate all the obstacles that hinder my development.
Overscheduling of child’s daily routine	27–31	Item 29: My father/mother does not allow me to have too much space to arrange my own activities.
Excessive care	32–36	Item 33: Whatever I want, my father/mother will try his best to satisfy me.
Excessive affective response	37–42	Item 39: When I encounter failures, my father/mother is unhappier than me.

### Data Analyses

We first conducted exploratory factor analyses to explore the factor structures of CPOS and CMOS. Principle axis factoring (PAF) approach with a direct oblimin rotation (δ = 0) was employed. To confirm the proposed factor structure, confirmatory factor analyses using AMOS 23.0 were performed to examine the factor structure of CPOS and CMOS, respectively. We used the values for the various goodness-of-fit indictors suggested by Hu and Bentler (1999) to assess the adequacy of models: comparative fit index (CFI) and Tucker Lewis Index (TLI) greater than 0.90 for an adequate model; Root Mean Square Error of Approximation (RMSEA) smaller than 0.06 for a good model fit, and between 0.06 and 0.08 for an acceptable model fit (Hu and Bentler, 1999). After testing the factor structure of CPOS and CMOS, respectively, we examined the hierarchical factor structure model of CPOS and CMOS. “Over-demandingness” was represented by “close monitoring,” “intrusion of child’s life and direction,” “overemphasis of child’s academic performance,” “frequent comparison of child’s achievement,” and “overscheduling of child’s daily routine,” while “over-responsiveness” was represented by “anticipatory problem solving,” “excessive care” and “excessive affective response.” Confirmatory factor analyses of the hierarchical factor models were performed. As suggested by Marsh and Shavelson (1985) that the goodness-of-fit for the higher order model cannot be better than that for the first-order model, as far as a higher order model fits the data, the higher order model is accepted as it provides a parsimonious factor structure for the construct.

To examine the factorial invariance of CPOS and CMOS across adolescent gender, multiple group analyses were performed. The mean and covariance structures analysis (MACS) approach was adopted in the study (Chen et al., 2005; Byrne and Stewart, 2006). Regarding CPOS, we first tested the configural invariance (i.e., no constraints were imposed; M7a). Then, the first-order factor loading invariance was tested (imposing equality constraints on first-order factor loadings; M7b). The two models (M7a and M7b) was compared, with non-significant chi-square difference value and change of CFI < 0.01 (Cheung and Rensvold, 2002) as indicators of model invariance. Next, the second-order factor loading invariance was tested (i.e., imposing equality constraints on first- and second-order factor loadings; M7c). Comparison of chi-square value and ΔCFI between M7b and M7c was performed, respectively. Then, we tested a series of nested models subsequently, including invariance of intercepts of measured variables (i.e., imposing equality constraints on first- and second-order factor loadings and intercepts of measured variables; M7d), invariance of intercepts of first-order latent factors (i.e., imposing equality constraints on first- and second-order factor loadings, and intercepts of measured variables and first-order factors; M7e), invariance of disturbances of first-order factors (i.e., imposing equality constraints on first- and second-order factor loadings, intercepts, and disturbances of first-order factors; M7f) and invariance of residual variance of observed variables (i.e., imposing equality constraints on first- and second-order factor loadings, intercepts, disturbances of first-order factors, and residual variances of measured variables; M7g) (Chen et al., 2005). Non-significant chi-square difference value and change of CFI < 0.01 (Cheung and Rensvold, 2002) were used as indicators in comparison of the nested models. Identical procedures were repeated to examine the factorial invariance of CMOS across adolescent gender.

## Results

### Exploratory Factor Analyses

The descriptive characteristics of the scales and subscales are listed in Table 2. Eigenvalues analyses showed that there were seven factors exceeding unity for CPOS and CMOS. For CPOS, “anticipatory problem solving” and “excessive care” were combined to form one factor, explaining 12.53% of the variance (Table 3), Besides, Item 36 loaded on “excessive affective response” instead of “excessive care.” For CMOS, combination of “intrusion of child’s life and direction” and “overscheduling of child’s daily routine” into one factor, explaining 34.83% of the variance (Table 4). Besides, Item 5 loaded on “close monitoring” instead of “intrusion of child’s life and direction.” The related factor loadings of CPOS and CMOS are shown in Tables 3, 4, respectively. However, as eigenvalues for the eighth factor were approached unity for both scales (0.99 and 0.91, respectively), we also examined whether an 8-factor model was represented in CPOS and CMOS, respectively. Exploratory factor analyses showed that an 8-factor model explained 59.97 and 63.57% of the total variance of CPOS and CMOS, respectively (Tables 3, 4). The patterns were identical to the conceptual model of overparenting.

**TABLE 2:** Descriptive statistics of Chinese paternal/maternal overparenting scales.

**Dimension**	**Item no.**	**Paternal overparenting**				**Maternal overparenting**			
		**Mean**	**SD**	**Skewness**	**Kurtosis**	**Mean**	**SD**	**Skewness**	**Kurtosis**
Close monitoring	1	3.92	1.45	–0.46	–0.62	4.33	1.48	–0.79	–0.23
	2	2.90	1.38	0.39	–0.59	3.65	1.51	–0.21	–0.84
	3	2.97	1.34	0.27	–0.67	3.62	1.50	–0.21	–0.85
	4	2.21	1.31	1.07	0.47	2.89	1.59	0.38	–0.97
Intrusion of child’s life and direction	5	2.56	1.35	0.66	–0.23	3.00	1.52	0.31	–0.86
	6	1.95	1.17	1.38	1.62	2.47	1.46	0.77	–0.36
	7	2.33	1.38	0.93	0.07	2.75	1.54	0.51	–0.78
	8	2.08	1.22	1.14	0.83	2.55	1.45	0.69	–0.41
	9	2.22	1.29	1.01	0.38	2.70	1.52	0.56	–0.71
	10	2.31	1.30	0.89	0.10	3.23	1.59	0.12	–1.06
Overemphasis of child’s academic performance	11	3.60	1.52	–0.18	–0.90	4.40	1.42	–0.77	–0.08
	12	2.93	1.51	0.38	–0.78	3.81	1.54	–0.27	–0.85
	13	2.21	1.29	0.96	0.31	3.09	1.56	0.27	–0.92
	14	2.64	1.50	0.65	–0.52	3.61	1.62	–0.12	–1.06
	15	2.42	1.45	0.86	–0.18	3.22	1.68	0.20	–1.17
Frequent comparison of child’s achievement with peers	16	2.65	1.69	0.72	–0.74	3.63	1.79	–0.14	–1.31
	17	2.20	1.40	1.13	0.40	3.29	1.76	0.16	–1.27
	18	2.13	1.32	1.20	0.77	2.75	1.58	0.55	–0.76
	19	2.45	1.46	0.79	–0.32	3.12	1.66	0.24	–1.12
	20	2.28	1.36	0.99	0.20	3.01	1.65	0.34	–1.06
Anticipatory problem solving	21	3.68	1.49	–0.31	–0.81	3.51	1.50	–0.13	–0.86
	22	3.04	1.42	0.14	–0.86	3.21	1.53	0.12	–0.95
	23	2.90	1.38	0.32	–0.61	3.12	1.49	0.20	–0.84
	24	3.17	1.44	0.11	–0.79	3.53	1.53	–0.09	–0.90
	25	3.13	1.40	0.11	–0.73	3.31	1.50	0.03	–0.88
	26	3.34	1.52	0.03	–0.93	3.43	1.56	–0.02	–0.96
Overscheduling of child’s daily routine	27	1.84	1.11	1.51	2.09	2.38	1.47	0.91	–0.08
	28	2.07	1.25	1.20	0.90	3.15	1.55	0.21	–0.97
	29	2.03	1.29	1.34	1.19	2.56	1.48	0.72	–0.41
	30	1.75	1.07	1.68	2.81	2.30	1.39	0.95	0.11
	31	1.87	1.14	1.44	1.83	2.26	1.31	0.92	0.18
Excessive care	32	2.72	1.38	0.42	–0.63	3.37	1.52	0.00	–0.92
	33	3.40	1.55	–0.04	–0.98	3.33	1.51	–0.01	–0.92
	34	2.26	1.36	0.97	0.17	2.43	1.49	0.85	–0.23
	35	3.07	1.56	0.19	–0.98	3.25	1.66	0.09	–1.16
	36	2.62	1.35	0.53	–0.41	2.73	1.44	0.44	–0.68
Excessive affective response	37	2.19	1.21	0.96	0.51	2.59	1.42	0.64	–0.37
	38	2.77	1.49	0.49	–0.67	3.23	1.56	0.08	–1.02
	39	2.63	1.42	0.63	–0.36	3.02	1.52	0.30	–0.82
	40	2.79	1.42	0.33	–0.77	3.08	1.51	0.20	–0.90
	41	2.62	1.38	0.51	–0.54	3.02	1.51	0.26	–0.86
	42	2.49	1.32	0.61	–0.32	2.86	1.48	0.39	–0.74

**TABLE 3:** Exploratory factor analyses models of Chinese Paternal Overparenting Scale.

**Item**	**Paternal overparenting**														
	**7-factor model with eigenvalues >1**							**8-factor model**							
1	–0.04	0.16	**0.56**	–0.09	0.03	–0.01	0.05	–0.04	0.10	**−0.54**	–0.13	0.06	0.03	0.04	0.10
2	0.01	–0.07	**0.87**	0.03	–0.03	0.01	–0.04	0.01	–0.03	**−0.85**	0.03	–0.06	0.03	–0.04	–0.03
3	0.07	0.01	**0.77**	–0.03	–0.04	–0.02	–0.02	0.06	0.06	**−0.76**	–0.01	–0.08	0.00	–0.01	–0.06
4	0.13	–0.07	**0.46**	0.09	–0.04	0.12	–0.21	0.13	–0.05	**−0.45**	0.08	–0.05	0.13	–0.21	0.02
5	0.00	–0.03	0.31	–0.05	0.00	0.16	**−0.41**	0.00	–0.06	–0.30	–0.10	0.03	0.14	**−0.42**	0.05
6	0.16	–0.05	0.08	0.04	–0.02	0.17	**−0.45**	0.16	–0.04	–0.08	0.02	–0.02	0.17	**−0.45**	0.01
7	0.01	–0.06	0.05	0.06	0.03	0.13	**−0.67**	0.01	–0.01	–0.06	0.06	0.01	0.14	**−0.66**	–0.06
8	0.11	0.08	–0.01	–0.06	–0.07	–0.03	**−0.74**	0.11	0.07	0.01	–0.09	–0.05	–0.05	**−0.73**	0.03
9	0.01	0.05	0.05	–0.08	–0.03	0.03	**−0.75**	0.02	0.04	–0.04	–0.12	–0.01	0.02	**−0.74**	0.00
10	0.15	0.11	0.10	–0.19	–0.04	0.01	**−0.41**	0.14	0.11	–0.09	–0.22	–0.03	–0.01	**−0.40**	–0.03
11	–0.08	0.10	0.12	**−0.56**	–0.13	0.18	–0.02	–0.09	0.01	–0.08	**−0.67**	–0.05	0.11	–0.02	0.06
12	0.07	0.16	0.04	**−0.62**	–0.11	0.10	–0.09	0.06	0.09	0.00	**−0.71**	–0.04	0.03	–0.08	0.02
13	0.37	0.04	0.05	**−0.38**	–0.08	0.12	0.01	0.35	0.04	–0.03	**−0.42**	–0.06	0.09	0.02	–0.03
14	0.14	0.02	0.11	**−0.55**	–0.15	0.09	–0.08	0.12	–0.02	–0.07	**−0.63**	–0.09	0.04	–0.07	–0.02
15	0.14	0.02	0.06	**−0.37**	–0.14	0.22	–0.13	0.12	0.00	–0.04	**−0.42**	–0.11	0.19	–0.13	–0.01
16	–0.01	–0.06	0.06	–0.11	0.02	**0.75**	0.07	–0.02	–0.01	–0.06	–0.10	–0.02	**0.75**	0.08	–0.07
17	0.16	0.01	0.01	–0.01	0.03	**0.76**	0.09	0.15	0.06	–0.02	0.01	–0.02	**0.77**	0.10	–0.04
18	0.04	–0.03	–0.05	0.06	–0.04	**0.83**	–0.04	0.03	–0.01	0.05	0.05	–0.06	**0.82**	–0.04	0.03
19	–0.07	0.04	0.03	–0.04	0.04	**0.73**	–0.14	–0.06	0.00	–0.03	–0.07	0.06	**0.71**	–0.15	0.08
20	0.02	0.08	0.00	–0.05	0.02	**0.66**	–0.16	0.02	0.04	0.00	–0.09	0.04	**0.63**	–0.17	0.08
21	–0.02	**0.73**	0.10	–0.02	0.07	–0.05	0.07	–0.04	**0.67**	–0.09	0.00	0.06	–0.04	0.08	0.10
22	0.02	**0.72**	0.02	–0.04	0.01	0.03	–0.11	0.00	**0.75**	–0.01	0.03	–0.05	0.05	–0.08	–0.03
23	0.05	**0.83**	–0.06	–0.04	0.05	0.05	–0.10	0.02	**0.85**	0.06	0.03	–0.01	0.08	–0.07	–0.01
24	0.08	**0.74**	0.03	–0.17	0.02	–0.04	0.01	0.06	**0.74**	–0.02	–0.13	–0.02	–0.03	0.04	–0.02
25	0.05	**0.76**	0.03	–0.06	–0.06	–0.06	–0.05	0.03	**0.78**	–0.02	0.00	–0.11	–0.04	–0.02	–0.02
26	0.02	**0.65**	0.04	0.00	–0.09	0.02	0.00	0.00	**0.59**	–0.03	0.02	–0.09	0.02	0.01	0.12
27	**0.75**	0.05	0.01	–0.06	0.01	0.03	0.00	**0.73**	0.05	–0.01	–0.09	0.02	0.03	–0.01	0.03
28	**0.49**	0.10	0.14	–0.03	0.02	0.06	–0.14	**0.48**	0.08	–0.13	–0.07	0.04	0.05	–0.15	0.07
29	**0.50**	–0.04	0.12	0.01	0.07	0.10	–0.20	**0.49**	0.00	–0.13	0.00	0.05	0.11	–0.20	–0.04
30	**0.78**	–0.01	0.02	0.01	–0.01	0.02	–0.01	**0.75**	0.01	–0.02	–0.01	–0.01	0.03	–0.02	0.02
31	**0.53**	0.00	0.02	0.01	–0.12	0.08	–0.03	**0.51**	0.01	–0.02	0.00	–0.12	0.08	–0.03	0.03
32	0.18	**0.36**	0.05	–0.06	–0.21	0.04	0.07	0.18	0.17	–0.03	–0.15	–0.09	–0.01	0.06	**0.34**
33	–0.10	**0.52**	0.02	0.10	–0.20	0.08	0.09	–0.10	0.24	–0.01	–0.01	–0.02	0.03	0.05	**0.54**
34	0.08	**0.29**	–0.03	0.18	–0.28	0.10	–0.05	0.09	–0.03	0.05	0.05	–0.06	0.05	–0.11	**0.62**
35	–0.05	**0.37**	0.06	0.12	–0.29	0.02	0.13	–0.04	–0.01	–0.06	–0.04	–0.04	–0.04	0.09	**0.71**
36	0.09	0.30	0.06	0.05	**−0.35**	0.00	0.01	0.09	0.10	–0.05	–0.04	–0.22	–0.04	–0.01	**0.38**
37	0.19	0.03	0.00	0.09	**−0.42**	0.09	–0.13	0.18	0.03	0.00	0.10	**−0.41**	0.10	–0.12	0.07
38	–0.04	0.04	0.01	–0.11	**−0.76**	0.01	–0.06	–0.07	0.06	0.00	–0.09	**−0.75**	0.01	–0.04	0.02
39	–0.03	–0.06	–0.03	–0.09	**−0.92**	–0.02	–0.06	–0.05	–0.03	0.03	–0.06	**−0.91**	–0.01	–0.04	0.01
40	–0.05	0.10	0.03	–0.05	**−0.76**	0.02	0.00	–0.07	0.11	–0.02	–0.01	**−0.76**	0.03	0.02	0.03
41	0.05	–0.01	0.05	–0.03	**−0.80**	–0.05	0.04	0.03	0.01	–0.05	0.00	**−0.78**	–0.04	0.06	0.04
42	0.10	0.01	0.06	–0.02	**−0.70**	0.00	0.04	0.08	0.03	–0.06	0.01	**−0.70**	0.01	0.06	0.03
Eigenvalue	14.72	5.67	1.88	1.45	1.33	1.19	1.05	14.72	5.67	1.88	1.45	1.33	1.19	1.05	0.99
% of Variance	34.11	12.53	3.54	2.52	2.29	1.85	1.47	34.13	12.59	3.57	2.54	2.33	1.88	1.49	1.45
Total variance (%)							58.31								59.97

*Bold values are the highest loadings obtained by a variable among the factors.*

**TABLE 4:** Exploratory factor analyses models of Chinese Maternal Overparenting Scale.

**Item**	**Maternal overparenting**														
	**7-factor model with eigenvalues >1**							**8-factor model**							
1	–0.06	–0.03	–0.05	–0.03	**0.53**	–0.19	0.10	–0.02	–0.05	–0.05	–0.01	**0.53**	–0.17	0.08	–0.01
2	–0.03	0.07	–0.02	0.02	**0.83**	–0.03	0.03	0.00	0.05	–0.02	0.04	**0.85**	0.02	0.00	0.01
3	0.00	0.06	–0.08	–0.01	**0.75**	–0.03	0.04	0.02	0.04	–0.09	0.01	**0.77**	0.01	0.01	0.03
4	0.23	0.07	0.03	0.13	**0.57**	0.08	0.01	0.28	0.07	0.04	0.10	**0.50**	0.08	0.01	0.08
5	0.26	0.04	0.02	0.18	**0.42**	–0.09	–0.01	**0.37**	0.06	0.05	0.12	0.33	–0.12	0.01	0.03
6	**0.39**	0.07	0.03	0.21	0.23	0.02	–0.04	**0.41**	0.08	0.05	0.14	0.14	–0.01	–0.02	0.14
7	**0.45**	–0.03	–0.03	0.24	0.29	0.07	–0.08	**0.62**	0.00	0.01	0.13	0.14	0.01	–0.04	0.05
8	**0.56**	–0.05	–0.12	0.15	0.21	0.05	–0.02	**0.78**	–0.01	–0.08	0.02	0.01	–0.03	0.04	0.05
9	**0.53**	–0.03	–0.13	0.18	0.23	0.01	–0.07	**0.75**	0.01	–0.08	0.05	0.03	–0.07	–0.02	0.04
10	**0.36**	–0.02	–0.13	0.05	0.15	–0.24	–0.03	**0.39**	–0.01	–0.10	–0.02	0.05	–0.28	0.00	0.10
11	–0.13	0.02	–0.01	0.20	0.10	**−0.64**	0.00	–0.01	0.03	0.00	0.17	0.08	**−0.67**	0.01	–0.12
12	0.11	0.01	–0.09	0.04	0.00	**−0.73**	0.01	0.04	0.01	–0.08	0.01	–0.01	**−0.76**	0.02	0.08
13	0.17	0.07	–0.07	0.17	0.08	**−0.37**	0.02	0.10	0.07	–0.06	0.14	0.07	**−0.38**	0.02	0.12
14	0.15	0.04	–0.07	0.02	0.06	**−0.67**	0.00	0.03	0.03	–0.06	0.01	0.05	**0.67**	0.00	0.13
15	0.15	0.08	0.02	0.23	0.04	**−0.47**	0.04	0.08	0.08	0.03	0.20	0.03	**−0.48**	0.05	0.12
16	–0.14	0.02	–0.01	**0.82**	0.04	–0.11	–0.04	–0.13	0.00	–0.03	**0.81**	0.10	–0.09	–0.06	0.01
17	–0.08	0.01	–0.03	**0.87**	0.00	–0.04	0.00	–0.11	–0.01	–0.05	**0.86**	0.06	–0.01	–0.02	0.06
18	0.09	0.05	0.00	**0.78**	–0.03	0.03	0.01	0.15	0.05	0.00	**0.71**	–0.04	0.01	0.02	0.04
19	0.07	–0.01	0.02	**0.78**	0.00	–0.07	0.05	0.20	0.00	0.02	**0.70**	–0.03	–0.10	0.07	–0.02
20	0.12	–0.03	–0.06	**0.72**	–0.01	–0.05	0.03	0.23	–0.01	–0.05	**0.64**	–0.05	–0.09	0.05	0.01
21	–0.07	–0.03	**−0.78**	0.02	0.05	0.02	0.06	–0.04	–0.04	**−0.78**	0.03	0.07	0.02	0.05	–0.03
22	0.01	0.02	**−0.84**	0.06	–0.01	0.04	0.00	0.01	0.01	**−0.84**	0.07	0.01	0.05	0.00	0.03
23	0.03	–0.03	**−0.91**	0.06	–0.02	0.05	0.00	0.03	–0.03	**−0.90**	0.06	0.00	0.05	0.00	0.03
24	0.01	0.05	**−0.75**	–0.07	–0.01	–0.12	0.03	0.00	0.05	**−0.74**	–0.06	0.00	–0.13	0.03	0.02
25	0.04	0.07	**−0.85**	–0.05	–0.05	–0.02	0.01	0.06	0.08	**−0.84**	–0.05	–0.05	–0.04	0.01	0.00
26	–0.02	0.13	**−0.75**	–0.04	0.02	–0.02	0.01	0.01	0.13	**−0.74**	–0.03	0.02	–0.02	0.01	–0.01
27	**0.67**	–0.01	–0.12	0.00	–0.01	–0.12	0.02	0.09	–0.05	–0.12	0.00	0.03	–0.07	–0.02	**0.66**
28	**0.41**	–0.03	–0.07	–0.04	0.30	–0.17	0.04	0.20	–0.04	–0.06	–0.05	0.28	–0.16	0.03	**0.30**
29	**0.57**	0.05	0.05	0.08	0.19	–0.02	–0.05	0.18	0.03	0.05	0.08	0.20	0.02	–0.08	**0.49**
30	**0.70**	0.05	0.01	0.00	–0.07	–0.11	0.06	–0.06	–0.02	0.00	0.01	0.00	–0.04	0.00	**0.86**
31	**0.50**	0.19	0.01	0.06	–0.10	–0.05	0.14	0.01	0.16	0.01	0.07	–0.05	–0.01	0.11	**0.53**
32	0.01	0.17	–0.10	–0.04	0.08	–0.17	**0.43**	–0.03	0.17	–0.10	–0.04	0.08	–0.17	**0.43**	0.03
33	–0.14	0.04	–0.14	–0.02	0.06	–0.04	**0.58**	–0.10	0.04	–0.14	–0.02	0.07	–0.04	**0.58**	–0.07
34	0.12	0.03	–0.03	0.11	–0.01	0.12	**0.67**	0.06	–0.03	–0.02	0.09	–0.02	0.12	**0.67**	0.09
35	–0.08	0.02	0.00	–0.06	0.04	–0.05	**0.76**	–0.02	0.02	0.01	–0.07	0.02	–0.06	**0.77**	–0.07
36	0.12	0.25	–0.04	–0.04	0.02	–0.06	**0.44**	0.04	0.25	–0.03	–0.05	0.01	–0.06	**0.44**	0.09
37	0.16	**0.56**	0.01	0.09	0.00	0.05	0.13	0.05	**0.56**	0.02	0.08	0.00	0.06	0.13	0.14
38	–0.05	**0.78**	–0.07	–0.01	0.06	–0.08	–0.02	–0.04	**0.78**	–0.07	–0.01	0.06	–0.08	–0.02	–0.02
39	–0.05	**0.92**	–0.02	–0.02	0.05	0.02	–0.02	0.00	**0.92**	–0.02	–0.02	0.04	0.02	–0.02	–0.04
40	–0.01	**0.86**	–0.04	–0.02	–0.01	–0.03	0.01	0.03	**0.86**	–0.04	–0.03	–0.03	–0.05	0.02	–0.03
41	–0.05	**0.87**	0.02	–0.01	0.00	0.00	0.02	–0.03	**0.87**	0.02	–0.01	0.00	–0.01	0.02	–0.03
42	0.05	**0.73**	–0.10	0.02	–0.03	0.04	0.02	–0.01	**0.72**	–0.10	0.02	–0.02	0.05	0.02	0.06
Eigenvalue	15.00	6.06	2.05	1.69	1.39	1.22	1.18	15.00	6.06	2.05	1.69	1.39	1.22	1.18	0.91
% of Variance	34.83	13.60	4.01	3.19	2, 49	1.96	1.86	34.87	13.63	4.05	3.25	2.53	2.02	1.88	1.35
Total variance (%)							61.94								63.57

*Bold values are the highest loadings obtained by a variable among the factors.*

To arrive at a more definitive answer on the dimensionality of the two scales, confirmatory factor analyses were performed with one-factor model, 7-factor model extracted from EFA of CPOS (i.e., combination of “anticipatory problem solving” and “excessive care” into one factor, and Item 36 loaded on “excessive affective response”), 7-factor model extracted from EFA of CMOS (i.e., combination of “intrusion of child’s life and direction” and “overscheduling of child’s daily routine” into one factor, and Item 5 belonging to “close monitoring”), and an 8-factor model reflecting the conceptualization of overparenting.

### Confirmatory Factor Analyses

Full information maximum likelihood (FIML) estimation was used to handle missing data (Arbuckle, 2007). As the skewness and kurtosis values of all items were less than 2 and 7, respectively (Table 2), the assumption of multivariate normality was supported (Curran et al., 1996). Hence, the maximum likelihood method was used in the confirmatory factor analysis.

Regarding CPOS, four models, including one-factor model (M1a), 7-factor model reflecting the 7-factor solution extracted from EFA of CPOS (M2a), 7-factor model reflecting the 7-factor solution extracted from EFA of CMOS (M3a), and 8-factor model (M4a) were tested. The results showed that one-factor model (M1a), 7-factor model reflecting the 7-factor solution extracted from EFA of CPOS (M2a), 7-factor model reflecting the 7-factor solution extracted from EFA of CMOS (M3a) did not fit well with the data, with values of CFI and TLI smaller than 0.90, respectively (Table 5). On the other hand, the 8-factor model of CPOS (M4a) showed an acceptable fit of the data, with values of CFI = 0.912 and TLI = 0.904 (>0.90; Hu and Bentler, 1999), and RMSEA = 0.054 (<0.06; Hu and Bentler, 1999; Table 5). However, large modification indices were found in five pairs of error covariances (Items 14 and 15; Items 19 and 20; Items 24 and 25; Items 25 and 26; Items 34 and 35) and each pair belonged to the same factor. These parameters were set to be free to obtain a better fit model (Byrne et al., 1989). The modified model (M5a) improved the goodness-of-fit indices, with values of CFI = 0.926 and TLI = 0.919 (>0.90; Hu and Bentler, 1999), and RMSEA = 0.050 (<0.06; Hu and Bentler, 1999; Table 5). Factor loadings of the items ranged from 0.54 to 0.88 (Table 6).

**TABLE 5:** Goodness of fit indices for confirmatory factor models and hierarchical factor models of Chinese paternal/maternal overparenting scales.

**Description**	**Model**	**Parent gender**	**χ^2^**	***df***	***x^2^/df***	**CFI**	**TLI**	**RMSEA**
One-factor model	1a	Paternal	21905.816^∗∗∗^	819	26.747	0.542	0.519	0.122
	1b	Maternal	27933.476^∗∗∗^	819	34.107	0.484	0.458	0.138
7-factor structure and pattern according to the EFA results of CPOS	2a	Paternal	5537.347^∗∗∗^	798	6.939	0.897	0.889	0.059
	2b	Maternal	6468.087^∗∗∗^	798	8.105	0.888	0.879	0.064
7-factor structure and pattern according to the EFA results of CMOS	3a	Paternal	5872.993^∗∗∗^	798	7.360	0.890	0.881	0.061
	3b	Maternal	6433.416^∗∗∗^	798	8.026	0.893	0.884	0.064
8-factor model – based on the conceptual framework	4a	Paternal	4841.745^∗∗∗^	791	6.121	0.912	0.904	0.054
	4b	Maternal	5666.098^∗∗∗^	791	7.163	0.907	0.899	0.060
8-factor model – With five pairs of error covariance correlated	5a	Paternal	4181.710^∗∗∗^	786	5.320	0.926	0.919	0.050
	5b	Maternal	4658.462^∗∗∗^	786	5.927	0.926	0.919	0.053
Hierarchical factor structure – two second-order	6a	Paternal	4644.099^∗∗∗^	805	5.769	0.917	0.911	0.052
	6b	Maternal	5108.791^∗∗∗^	805	6.346	0.918	0.912	0.056

**^∗∗∗^p* < 0.001.*

**TABLE 6:** Standardized factor loadings of 8-factor structure model and hierarchical factor models of Chinese paternal/maternal overparenting scales.

**Higher-order construct**	**Construct**	**Item**	**8-factor structure model**		**Hierarchical factor structure model**			
			**Paternal**	**Maternal**	**Paternal**		**Maternal**	
			**overparenting**	**overparenting**	**overparenting**		**overparenting**	
			**Factor**	**Factor**	**First order**	**Second order**	**First order**	**Second order**
			**loading**	**loading**	**factor loading**	**factor loading**	**factor loading**	**factor loading**
**Over-demandingness**								
	Close monitoring					0.73		0.77
		1	0.58	0.61	0.57		0.60	
		2	0.85	0.88	0.85		0.88	
		3	0.84	0.85	0.83		0.84	
		4	0.66	0.72	0.66		0.72	
	Intrusion of child’s life and direction					0.89		0.91
		5	0.72	0.74	0.71		0.74	
		6	0.71	0.68	0.70		0.68	
		7	0.72	0.79	0.71		0.79	
		8	0.81	0.83	0.82		0.83	
		9	0.83	0.87	0.83		0.87	
		10	0.69	0.65	0.70		0.66	
	Overemphasis of child’s academic performance					0.84		0.80
		11	0.68	0.67	0.66		0.66	
		12	0.78	0.78	0.77		0.78	
		13	0.74	0.71	0.74		0.71	
		14	0.82	0.82	0.83		0.82	
		15	0.78	0.76	0.79		0.79	
	Frequent comparison of child’s achievement with peers					0.82		0.78
		16	0.71	0.74	0.71		0.74	
		17	0.77	0.78	0.77		0.78	
		18	0.82	0.80	0.82		0.80	
		19	0.83	0.89	0.83		0.89	
		20	0.83	0.87	0.83		0.87	
	Overscheduling of child’s daily routine					0.88		0.86
		27	0.79	0.77	0.79		0.77	
		28	0.76	0.70	0.76		0.71	
		29	0.75	0.75	0.75		0.75	
		30	0.77	0.76	0.77		0.76	
		31	0.64	0.59	0.64		0.58	
**Over-responsiveness**								
	Anticipatory problem solving					0.84		0.82
		21	0.66	0.76	0.66		0.76	
		22	0.72	0.80	0.72		0.80	
		23	0.81	0.85	0.81		0.85	
		24	0.83	0.86	0.83		0.85	
		25	0.88	0.91	0.88		0.91	
		26	0.75	0.86	0.75		0.86	
	Excessive care					0.91		0.87
		32	0.67	0.72	0.67		0.72	
		33	0.67	0.69	0.66		0.69	
		34	0.59	0.61	0.59		0.61	
		35	0.65	0.72	0.64		0.71	
		36	0.68	0.70	0.69		0.70	
	Excessive affective response					0.84		0.84
		37	0.54	0.67	0.54		0.67	
		38	0.79	0.82	0.79		0.82	
		39	0.86	0.89	0.86		0.89	
		40	0.85	0.90	0.85		0.90	
		41	0.82	0.86	0.82		0.86	
		42	0.79	0.80	0.79		0.80	

Identical factor models were tested for CMOS (M1b, M2b, M3b and M4b). Similar patterns were found in CMOS. The results showed that one-factor model (M1b), 7-factor model reflecting the 7-factor solution extracted from EFA of CPOS (M2b), 7-factor model reflecting the 7-factor solution extracted from EFA of CMOS (M3b) did not fit well with the data, with values of CFI and TLI smaller than 0.90, respectively (Table 5). The 8-factor model of CMOS (M4b) showed a marginal fit of the data, with values of CFI = 0.907, TLI = 0.899, and RMSEA = 0.060. After freeing the five pairs of error covariances (Items 14 and 15; Items 19 and 20; Items 24 and 25; Items 25 and 26; Items 34 and 35), the modified model (M5b) showed a good fit of the data, with values of CFI = 0.926 and TLI = 0.919 (>0.90; Hu and Bentler, 1999), and RMSEA = 0.053 (<0.06; Hu and Bentler, 1999; Table 5). Factor loadings of the items ranged from 0.59 to 0.91 (Table 6). Hence, the findings supported an 8-factor structure in CPOS and CMOS, respectively.

Regarding CPOS, the 8 dimensions were correlated with each other, with Pearson’s r ranged from 0.18 to 0.70 (Table 7). However, the dimensions of paternal “close monitoring,” “intrusion of child’s life and direction,” “overemphasis of child’s academic performance,” “frequent comparison of child’s achievement,” and “overscheduling of child’s daily routine” were highly correlated with each other, with Pearson’s r ranged from 0.47 to 0.70, but were moderately correlated with dimensions of “anticipatory problem solving” (Pearson’s r ranged from 0.22 to 0.42), “excessive care” (Pearson’s r ranged from 0.18 to 0.37) and “excessive affective response” (Pearson’s r ranged from 0.31 to 0.50). At the same time, the three dimensions (anticipatory problem solving, excessive care and excessive affective response) were highly correlated with each other, with Pearson’s r ranged from 0.63 to 0.65 (Table 7). Similar patterns were also found in CMOS. All correlation coefficients among the 8 dimensions were significant, with Pearson’s r ranged from 0.14 to 0.72 (Table 7). Besides, the dimensions of maternal “close monitoring,” “intrusion of child’s life” and direction, “overemphasis of child’s academic performance,” “frequent comparison of child’s achievement,” and “overscheduling of child’s daily routine” were highly correlated with each other, with Pearson’s r ranged from 0.51 to 0.72, but showed small to moderate correlations with dimensions of “anticipatory problem solving” (Pearson’s r ranged from 0.22 to 0.48), “excessive care” (Pearson’s r ranged from 0.14 to 0.35) and “excessive affective response” (Pearson’s r ranged from 0.22 to 0.39). Likewise, the three dimensions (anticipatory problem solving, excessive care and excessive affective response) were highly correlated with each other, with Pearson’s r ranged from 0.61 to 0.66 (Table 7). The patterns of inter-factor correlations suggested the existence of second-order factors (Gorsuch, 1983). As such, a hierarchical factor structure of the CPOS and CMOS was tested, respectively.

**TABLE 7:** Correlations of Chinese paternal/maternal overparenting scales and their subscales.

					**Cronbach’s**												
	**Mean**		***S.D.***		**Alpha**		**Correlations**										
	**P**	**M**	**P**	**M**	**P**	**M**	**1**	**2**	**3**	**4**	**5**	**6**	**7**	**8**	**9**	**10**	**11**
Overparenting	2.60	3.11	0.80	0.91	0.95	0.96		0.90	0.79	0.74	0.77	0.79	0.69	0.75	0.73	0.62	0.69
Over-demandingness	2.42	3.11	0.90	1.05	0.95	0.95	0.90		0.43	0.78	0.90	0.82	0.83	0.82	0.43	0.30	0.37
Over-responsiveness	2.87	3.12	0.99	1.10	0.93	0.94	0.80	0.46		0.41	0.32	0.47	0.24	0.40	0.88	0.84	0.89
Close monitoring	3.00	3.62	1.10	1.25	0.81	0.84	0.71	0.75	0.43		0.67	0.59	0.51	0.58	0.41	0.31	0.32
Intrusion of child’s life and direction	2.24	2.78	1.01	1.21	0.88	0.89	0.73	0.88	0.29	0.60		0.62	0.67	0.72	0.33	0.21	0.28
Overemphasis of child’s academic performance	2.76	3.63	1.19	1.27	0.88	0.87	0.82	0.83	0.52	0.58	0.62		0.61	0.56	0.48	0.35	0.39
Frequent comparison of child’s achievement with peers	2.34	3.16	1.22	1.47	0.90	0.92	0.70	0.84	0.28	0.47	0.68	0.61		0.56	0.25	0.14	0.22
Overscheduling of child’s daily routine	1.91	2.53	0.94	1.12	0.86	0.84	0.76	0.83	0.41	0.54	0.70	0.61	0.64		0.36	0.30	0.37
Anticipatory problem solving	3.21	3.35	1.18	1.32	0.90	0.94	0.71	0.40	0.89	0.42	0.24	0.48	0.22	0.34		0.61	0.63
Excessive care	2.81	3.02	1.06	1.16	0.79	0.82	0.64	0.32	0.85	0.32	0.18	0.37	0.18	0.30	0.65		0.66
Excessive affective response	2.58	2.97	1.13	1.29	0.90	0.93	0.74	0.46	0.87	0.38	0.31	0.50	0.31	0.42	0.63	0.64	

*All correlation coefficients are significant with *p* < 0.001. P, Paternal; M, Maternal. The coefficients above the diagonal are correlations among the scale and corresponding subscales of maternal overparenting, and those below the diagonal are correlations among the scale and corresponding subscales of paternal overparenting.*

### Hierarchical Factor Analysis on Second-Order Factor Models

Based on the theoretical framework of parenting style (Maccoby and Martin, 1983) and the conceptualization of overparenting (Locke et al., 2012), it is expected that two qualitatively distinctive dimensions (over-demandingness and over-responsiveness) exist in overparenting. Hence, two higher-order latent constructs (i.e., over-demandingness and over-responsiveness) were added to the tested model. While “over-demandingness” was represented by “close monitoring,” “intrusion of child’s life and direction,” “overemphasis of child’s academic performance,” “frequent comparison of child’s achievement” and “overscheduling of child’s daily routine,” “over-responsiveness” was represented by “anticipatory problem solving,” “excessive care” and “excessive affective response.” Regarding CPOS, the hierarchical factor structure (M6a) showed a good fit of the data, with CFI and TLI values of 0.917 and 0.911, respectively (>0.90; Hu and Bentler, 1999), and RMSEA value of 0.052 (<0.06; Hu and Bentler, 1999; Table 5). The dimensions (close monitoring, intrusion of child’s life, overemphasis of child’s academic performance, frequent comparison of child’s achievement and overscheduling of child’s daily routine) represented “paternal over-demandingness” well, explaining 58.2% of the variance. The second-order factor loadings ranged from 0.73 to 0.89 (Table 6). The three primary factors (anticipatory problem solving, excessive care and excessive affective response) also corresponded to “paternal over-responsiveness,” explaining 43.2% of the variance. The second-order factor loadings ranging from 0.84 to 0.91 (Table 6). For CMOS, the hierarchical factor structure (M6b) fitted the data well, with CFI, and TLI values of 0.918 and 0.912, respectively (>0.90; Hu and Bentler, 1999), and RMSEA value of 0.056 (<0.06; Hu and Bentler, 1999; Table 5). The factors fell into the corresponding hierarchical factors of “over-demandingness” and “over-responsiveness,” with second-order factor loadings ranging from 0.77 to 0.91, and from 0.82 to 0.87, respectively. The 5 factors (close monitoring, intrusion of child’s life, overemphasis of child’s academic performance, frequent comparison of child’s achievement and overscheduling of child’s daily routine) explained 45.2% of the variance of “over-demandingness,” and the three factors (anticipatory problem solving, excessive care and excessive affective response) explained 47.6% of the variance of “over-responsiveness.”

**FIGURE 1:**
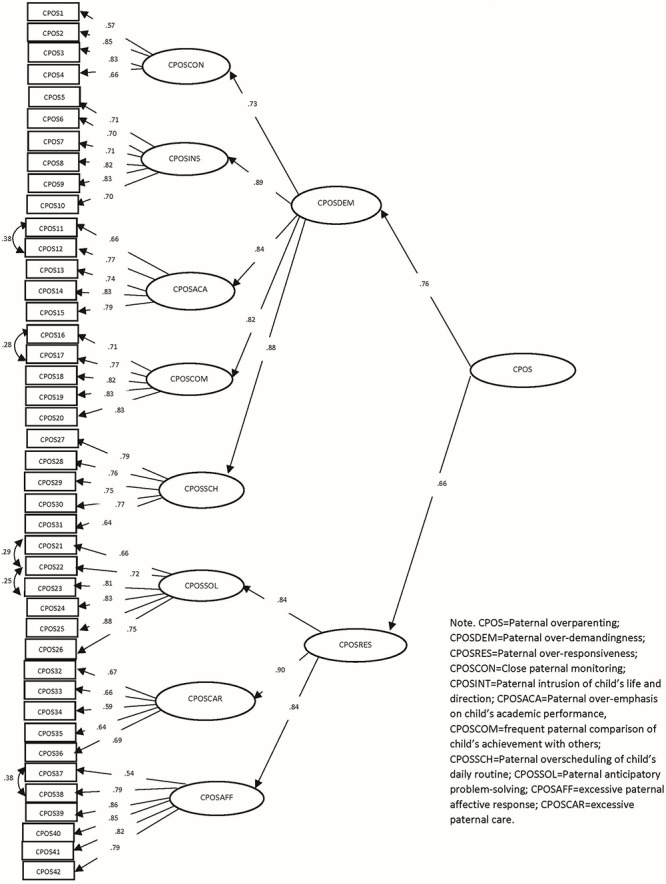
Factor structure and standardized coefficients of chinese Paternal Overparenting Scale.

**FIGURE 2:**
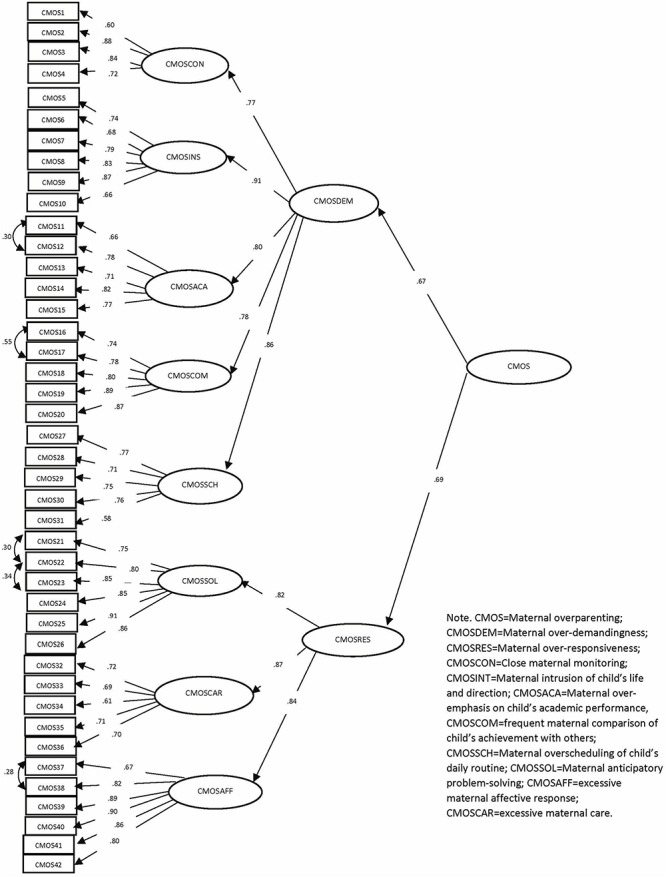
Factor structure and standardized coefficients of chinese Maternal Overparenting Scale.

There were some characteristics of two higher-order factor models of CPOS and CMOS. First, the five factors of “close monitoring,” “intrusion of child’s life and direction,” “overemphasis of child’s academic performance,” “frequent comparison of child’s achievement with others” and “overscheduling of child’s daily routine” corresponded well to the latent construct of “over-demandingness,” and the three factors of “anticipatory problem solving,” “excessive care” and “excessive affective response” reflected “over-responsiveness,” contributing to a respectable amount of variances of the two second-order latent constructs of the measurements, respectively. Second, the factor loadings of the first-order factors corresponding to “over-demandingness” and “over-responsiveness” were high, supporting the hierarchical factor structure in both CPOS and CMOS (Table 6). Third, the two second-order latent constructs of “over-demandingness” and “over-responsiveness” were qualitatively distinctive, but they correlated with each other reasonably in CPOS and CMOS, respectively (Table 7). These observations are in agreement with the proposed conceptualization intrinsic to the model. Figures 1, 2 showed the hierarchical factor structures of CPOS and CMOS, respectively.

### Invariance Tests of Hierarchical Factor Models Across Adolescent Gender

Multiple group analyses were performed to examine whether there was invariance across adolescent boys and girls on the hierarchical factor models of CPOS and CMOS, respectively. Regarding the hierarchical factor model of CPOS, the unconstrained model (M7a) showed a good fit of the data, with value of CFI = 0.909 (>0.90; Hu and Bentler, 1999) and RMSEA = 0.039 (<0.06; Hu and Bentler, 1999; Table 8), indicating that the factor pattern was invariant across adolescent gender. When configural invariance (M7a) was assumed, we then tested the first-order factor loading invariance (i.e., M7b). It is not uncommon that Chi-square difference value was significant between two groups (Δx^2^ = 97.322, *p* < 0.001), as the likelihood ratio test is sensitive to large sample size (Byrne, 2001). The change of CFI value between two groups was 0.002, supporting first-order factor loading invariance across adolescent gender (ΔCFI < 0.01; Cheung and Rensvold, 2002). When first-order factor loading invariance was supported, we tested second-order factor loading invariance across adolescent gender (M7c). The change of CFI between M7b and M7c was 0.000 (ΔCFI < 0.01; Cheung and Rensvold, 2002), supporting the second-order factor loadings invariance of CPOS. The results also showed invariance of intercepts of measured variables (M7d), with ΔCFI between M7c and M7d was 0.002. Invariance of intercepts of first-order factors (M7e) was also supported, with ΔCFI between M7d and M7e was 0.002. There was invariance on the disturbances of first-order factors (ΔCFI between M7e and M7f = 0.001). However, there was difference between boys and girls in residual variances of measured variables (M7g), with ΔCFI = 0.011 between M7g and M7f (ΔCFI > 0.01; Cheung and Rensvold, 2002). As suggested by Widaman and Reise (1997), invariance of factor loadings and intercepts are more relevant in assessing factorial invariance of a measurement between different groups. Hence, the hierarchical factor model of CPOS was considered as invariant between boys and girls (Table 8).

**TABLE 8:** Goodness of fit indices for factorial invariance of second-order factor model of Chinese paternal/maternal overparenting scales across adolescent gender.

**Scale**	**Model**	**Description**	***x*^2^**	***df***	**CFI**	**RMSEA**	**Comparison**	**Δx^2^**	**ΔCFI**	**Δdf**
Paternal Overparenting	M7a	Baseline model (i.e., configural invariance)	5854.172^∗∗∗^	1610	0.909	0.039				
	M7b	First-order factor loadings invariant	5951.494^∗∗∗^	1644	0.907	0.039	M7b and M7a	97.322^∗∗∗^	0.002	34
	M7c	First- and second-order factor loadings invariant	5968.014^∗∗∗^	1650	0.907	0.039	M7c and M7b	16.520^*^	0.000	6
	M7d	First- and second-order factor loadings and intercepts of measured variables invariant	6103.757^∗∗∗^	1684	0.905	0.039	M7d and M7c	135.743^∗∗∗^	0.002	34
	M7e	First- and second-order factor loadings, and intercepts of measured variables and first-order factors invariant	6204.894^∗∗∗^	1692	0.903	0.039	M7e and M7d	101.137^∗∗∗^	0.002	8
	M7f	First-and second-order factor loadings, intercepts, and disturbances of first-order factors invariant	6257.328^∗∗∗^	1703	0.902	0.039	M7f and M7e	52.434^∗∗∗^	0.001	11
	M7g	First-and second-order factor loadings, intercepts, disturbances of first-order factors, and residual variances of measured variables invariant	6813.195^∗∗∗^	1750	0.891	0.041	M7g and M7f	554.867^∗∗∗^	0.011	47
Maternal Overparenting	M8a	Baseline model (i.e., configural invariance)	6283.517^∗∗∗^	1610	0.912	0.041				
	M8b	First-order factor loadings invariant	6318.738^∗∗∗^	1644	0.912	0.041	M8b and M8a	35.221	0.000	34
	M8c	First- and second-order factor loadings invariant	6324.250^∗∗∗^	1650	0.912	0.040	M8c and M8b	5.512	0.000	6
	M8d	First- and second-order factor loadings and intercepts of measured variables invariant	6443.479^∗∗∗^	1684	0.910	0.040	M8d and M8c	119.229^∗∗∗^	0.002	34
	M8e	First- and second-order factor loadings, and intercepts of measured variables and first-order factors invariant	6547.180^∗∗∗^	1692	0.909	0.041	M8e and M8d	103.702^∗∗∗^	0.001	8
	M8f	First-and second-order factor loadings, intercepts, and disturbances of first-order factors invariant	6561.978^∗∗∗^	1703	0.908	0.041	M8f and M8e	14.798	0.001	11
	M8g	First-and second-order factor loadings, intercepts, disturbances of first-order factors, and residual variances of measured variables invariant	6837.641^∗∗∗^	1750	0.904	0.041	M8g and M8f	275.664^∗∗∗^	0.005	47

^*^p < 0.05, *^∗∗∗^p* < 0.001.

For hierarchical factor model of CMOS, the model showed configural invariance (CFI = 0.912, RMSEA = 0.041; M8a), first-order factor loadings invariance (Δ*x*^2^ = 35.221, *p* > 0.05; ΔCFI = 0.000; between M8a and M8b), second-order factor loadings invariance (Δ*x*^2^ = 5.512, *p* > 0.05;ΔCFI = 0.000; between M8b and M8c), invariance in intercepts of measured variables (ΔCFI = 0.002; between M8c and M8d), invariance in intercepts of first-order factors (ΔCFI = 0.001; between M8d and M8e), invariance in disturbances of first-order factors (Δ*x*^2^ = 14.798, *p* > 0.05;ΔCFI = 0.001; between M8e and M8f) and invariance in residual variances of measured variables (ΔCFI = 0.005; between M8f and M8g) (Table 8), suggesting that there was invariance in the hierarchical factor models of CMOS across adolescent gender (Table 8; Chen et al., 2005).

### Internal Consistency

Both CPOS and CMOS showed good internal consistency, with Cronbach’s alpha values of 0.95 and 0.96, respectively (Table 7). The Cronbach’s alpha values of paternal over-demandingness subscale, maternal over-demandingness subscale, paternal over-responsiveness subscale and maternal over-responsiveness subscale were 0.95,0.95, 0.93, and 0.94, respectively. The first-order subscales also showed good internal consistency, with Cronbach’s alpha values ranging from 0.79 to 0.94. Table 7 shows the Cronbach’s alpha values of the measures and their subscales.

## Discussion

The study examined the dimensionality of Chinese Paternal (Maternal) Overparenting Scale (CPOS and CMOS) and factorial invariance across adolescent gender. Though overparenting has blossomed rapidly in both local and international contexts (Gibbs, 2009; Leung and Busiol, 2016), previous researches constrained their focus on studying the impacts of overparenting in emerging adults and little is known about the influences of overparenting in early adolescents. The lack of validated instruments in assessing overparenting in early adolescents has hindered the research development particularly in the Chinese culture. The results of exploratory factor analysis showed that a 7-factor solution was identified by CPOS, with “anticipatory problem solving” and “excessive care” forming one factor. As fathers are more involved in fulfilling the instrumental needs of their children (Spence, 1993), anticipatory problem solving is the manifestation of paternal care and support for their children. In contrast, “intrusion of child’s life and direction” and “overscheduling of child’s daily routine” were combined to form one factor for CMOS. As mothers are mainly responsible for providing daily care and monitoring of their children (Kluwer et al., 2000), overscheduling of their children’s daily routine is a demonstration of maternal intrusion into their children’s daily routine and developmental direction.

In this study, an 8-factor structure of Chinese overparenting was identified in young adolescents by confirmatory factor analyses, including “close monitoring,” “intrusion of child’s life and direction,” “over-emphasis on child’s academic performance,” “frequent comparison of child’s achievement with others,” “overscheduling of child’s daily routine,” “anticipatory problem-solving,” “excessive affective response” and “excessive care,” resembling the factor structure identified in emerging adults (Leung et al., 2018). The present findings suggest that the concepts of Chinese overparenting is also applied to early adolescents. In early adolescence, individuals strive for greater independence and autonomy in their developmental paths. Indeed, parents may need to modify their parenting styles so that their children can have greater space for exploration and development (Steinberg and Morris, 2001). However, those parents who exercise overparenting fail to grant more autonomy for their children to learn from trials and errors. Instead, they intrude into the daily routine and life direction of their children and “mow” away any obstacles that appear in their life paths so that they can protect their children from risks and ensure the future “success” of their children (Padilla-Walker and Nelson, 2012; Segrin et al., 2012; Garst and Gagnon, 2015). The present study suggests that overparenting behavior exist in early adolescence as well as emerging adulthood.

Moreover, hierarchical factor analyses showed the existence of second-order factors of “over-demandingness” and “over-responsiveness” in CPOS and CMOS, respectively, corresponding to the conceptualization of parenting style suggested by Maccoby and Martin (1983). There were debates concerning whether overparenting is the manifestation of “excessive” parental demandingness and responsiveness (Locke et al., 2012), or it embraces different distinctive features that differentiate overparenting from other parenting practice (Segrin et al., 2012). More queries were raised on how the concepts of overparenting were linked with the existing literature of parenting style, parental control and parental support (Locke et al., 2012; Segrin et al., 2013; Leung et al., 2018). This study provides important cues on the conceptualization of overparenting because different distinctive features of Chinese overparenting (Leung et al., 2018) fit the dimensions of “over-demandingness” and “over-responsiveness” well. While “parental over-demandingness” comprises “close monitoring,” “intrusion of child’s life and direction,” “over-emphasis on child’s academic performance,” “frequent comparison of child’s achievement with others,” and “overscheduling of child’s daily routine”, “parental over-responsiveness” includes “anticipatory problem-solving,” “excessive affective response,” and “excessive care.” In other words, “parental over-demandingness” entails parental intrusion and over-emphasis of children’s achievement, whereas “parental over-responsiveness” embodies parental over-involvement and over-protection on child’s daily needs and affection. The identification of second-order constructs of “over-demandingness” and “over-responsiveness” provides a refined conceptual foundation for overparenting, which enriches the conceptualization of overparenting. Practically, the subscales of over-demandingness and over-responsiveness can help family practitioners identify families exercising the extreme parenting styles and provide necessary service to assist the families.

Furthermore, the hierarchical factor models of CPOS and CMOS were found invariant across adolescent gender, suggesting that adolescent boys and girls shared similar interpretations about the characteristics and patterns of overparenting. Though previous literature suggested that adolescent girls were more sensitive to maternal affection and authoritarian parenting style than were boys (Radziszewska et al., 1996; Shek, 2008), the findings indicated that they perceive paternal and maternal overparenting from a similar framework. This may partly be explained by the fact that Chinese parents emphasize academic excellence in boys and girls (Chao and Sue, 1996). This is important to assess factorial invariance of CPOS and CMOS to ensure the congruence of the measurements between gender groups. The results showed that CPOS and CMOS are applicable to assess paternal and maternal overparenting across adolescent gender, which facilitates further research on overparenting on adolescent development.

Apart from the theoretical and practical implications, there are also methodological implications in examining hierarchical factor models. First, it offers a parsimonious structure on how first-order factors are interrelated into meaningful patterns (Chen et al., 2005). Besides, hierarchical factor analysis removes random measurement error of the first-order factors and suggests the variance of the second-order factor to be explained by the first-order factors (Brown, 2006). The findings also fit nicely into the bi-dimensional model based on parental over-demandingness and parental over-responsiveness. In summary, CPOS and CMOS show good psychometric properties that can be used to assess overparenting in young Chinese adolescents, which encourage more researchers to conduct overparenting researches on early adolescent samples.

There are several limitations of the study. First, the study employed a sample of young adolescents without taking the perspective of parents in the study. Though it is justified to collect the views of adolescents as they are the “receivers” and “critical observers” of parenting practice (Casas, 2011; Elstad and Stefansen, 2014), multiple sources of data would give us a more comprehensive picture on how overparenting can be conceptualized and operationalized. Second, though the two second-order factors of “over-demandingness” and “over-responsiveness” were identified in the hierarchical factor models of both CPOS and CMOS, there is a need to examine the convergent validity of over-demandingness and over-responsiveness with measures of parenting style. Third, although there are views that residual invariance is less relevant in testing factorial invariance of a measurement tool between groups (Widaman and Reise, 1997), further investigations on the difference of residuals of measured variables of CPOS are suggested. Fourth, the study was conducted in a sample of young Chinese adolescents in Hong Kong. It is recommended to replicate the study in other Chinese communities (e.g., American Chinese, Chinese in Mainland China and Taiwan etc.) and perhaps some Asian countries sharing similar cultural and social characteristics (e.g., Japan, South Korea).

In summary, the hierarchical factor models of CPOS and CMOS showed that there were eight first-order factors (close monitoring, intrusion of child’s life and direction, over-emphasis on child’s academic performance, frequent comparison of child’s achievement with others, overscheduling of child’s daily routine, anticipatory problem-solving, excessive affective response and excessive care) which can be subsumed under two second-order factors of “over-demandingness” and “over-responsiveness.” The findings provide support for the conceptual framework of overparenting (Segrin et al., 2012; Leung et al., 2018) and at the same time support the conceptualization of overparenting as excessive “demandingness” and “responsiveness” to their children (Maccoby and Martin, 1983; Locke et al., 2012). Moreover, both CPOS and CMOS showed good internal consistency and factorial invariance across adolescent gender. The measures showed good psychometric properties that are adequate to assess overparenting in young Chinese adolescents. In view of the strong need for more comprehensive conceptualization of overparenting but a dearth of validated instruments for measuring overparenting in early adolescents, this study takes a humble step to contribute to the limited scientific literature on overparenting in early adolescence.

## Data Availability

The datasets generated for this study are available on request to the corresponding author.

## Ethics Statement

This study was approved by the Human Subjects Ethics Sub-committee (HSESC) (or its Delegate) of The Hong Kong Polytechnic University. The respondents and their parents had given written informed consent before data collection.

## Author Contributions

JL conceived the study, participated in the design and coordination, interrupted the data, and drafted the manuscript. DS participated in consultation of the research design, interpreted the data, and edited the manuscript. All authors read and approved the manuscript.

## Conflict of Interest Statement

The authors declare that the research was conducted in the absence of any commercial or financial relationships that could be construed as a potential conflict of interest.
